# Spin Biochemistry Modulates Reactive Oxygen Species (ROS) Production by Radio Frequency Magnetic Fields

**DOI:** 10.1371/journal.pone.0093065

**Published:** 2014-03-28

**Authors:** Robert J. Usselman, Iain Hill, David J. Singel, Carlos F. Martino

**Affiliations:** 1 Electromagnetics Division, National Institute of Standards and Technology, Boulder, Colorado, United States of America; 2 Department of Chemistry and Biochemistry, Montana State University, Bozeman, Montana, United States of America; 3 Department of Mechanical Engineering, University of Nevada Reno, Reno, Nevada, United States of America; German Cancer Research Center, Germany

## Abstract

The effects of weak magnetic fields on the biological production of reactive oxygen species (ROS) from intracellular superoxide (O_2_^•−^) and extracellular hydrogen peroxide (H_2_O_2_) were investigated *in vitro* with rat pulmonary arterial smooth muscle cells (rPASMC). A decrease in O_2_^•−^ and an increase in H_2_O_2_ concentrations were observed in the presence of a 7 MHz radio frequency (RF) at 10 μT_RMS_ and static 45 μT magnetic fields. We propose that O_2_^•−^ and H_2_O_2_ production in some metabolic processes occur through singlet-triplet modulation of semiquinone flavin (FADH^•^) enzymes and O_2_^•−^ spin-correlated radical pairs. Spin-radical pair products are modulated by the 7 MHz RF magnetic fields that presumably decouple flavin hyperfine interactions during spin coherence. RF flavin hyperfine decoupling results in an increase of H_2_O_2_ singlet state products, which creates cellular oxidative stress and acts as a secondary messenger that affects cellular proliferation. This study demonstrates the interplay between O_2_^•−^ and H_2_O_2_ production when influenced by RF magnetic fields and underscores the subtle effects of low-frequency magnetic fields on oxidative metabolism, ROS signaling, and cellular growth.

## Introduction

One of the greatest challenges in the field of chemical and physical biology is to bridge the knowledge gap between the atomic level and the cellular level [1]. Focused at the biological quantum/classical interface, an emerging field called quantum biology has promised to offer new and compelling insights into fundamental underlying cellular processes from the perspective of quantum phenomena [2], [3]. Following this paradigm, we present a novel methodology for indirectly investigating possible quantum effects in biological systems by applied static and alternating magnetic fields that induce changes in magnetically sensitive free radical pairs in biochemical reactions. Some evidence shows the effects of such exposures on cellular morphology, growth curves, and protein expression, implying an underlying metabolic influence [4]–[7]. The effect of weak magnetic fields on cellular metabolic processes is not well understood and little is known about how magnetic fields influence reaction rates in oxidative metabolism [8]–[10]. This work aims to elucidate biological responses that are sensitive to radio frequency (RF) magnetic fields involving the production of reactive oxygen species (ROS), of which are born presumably from spin-correlated free radical pairs.

In many biological processes, the reactivity of molecular oxygen and the formation of oxygen radical intermediates are a consequence of oxidative respiration [11], [12]. Most organisms have developed protective enzyme systems that mediate ROS products. One of the first steps in ROS production is the one-electron reduction of molecular oxygen (O_2_) that results in the formation of superoxide (O_2_^•−^). Superoxide is often a precursor for other ROS species such as hydrogen peroxide (H_2_O_2_), peroxynitrite (ONOO^−^), lipid and hydroxyl radicals (^•^OH) [13], where over-production of O_2_^•−^ often leads to oxidative stress. However, under normal physiological conditions, the role of O_2_^•−^ is beginning to emerge as an important signaling molecule that controls specific biochemical reactions and metabolic pathways [12]. The central tenet of this paradigm is that many cellular processes are capable of producing O_2_^•−^, whereas most H_2_O_2_ generation is formed exclusively from a O_2_^•−^ precursor. The link between O_2_^•−^ consumption and H_2_O_2_ production can involve a reduced flavin enzyme that transfers an electron to activate molecular oxygen into O_2_^•−^, which is then either released or enzymatically converted into H_2_O_2_ [11], [14]. In principle, this reaction could include a magnetically sensitive spin-correlated radical pair between a flavin semiquinone (FADH^•^) and O_2_^•−^ that can be mediated by weak magnetic fields [15]–[17].

A major challenge in modern bio-electromagnetics is to elucidate molecular mechanisms and interactions between biological systems and electromagnetic fields [18]. The spin-correlated radical-pair mechanism (SCRPM) offers the most plausible explanation on how magnetic fields might influence biochemical reactions [8], [19], [20]. Most notably, the excitonic energy transfer in photosynthesis has been well-described by the SCRPM [21], [22]. More recently, SCRPM is speculated to play a role in magnetoreception, which includes bird [23] and fruit fly navigation [24], vitamin B_12_-dependent enzymes [20], and ATP production [25]. In one form or another, magnetoreceptors are thought to be present in organisms ranging from magnetotactic and photosynthetic bacteria to insects, birds, and mammals, albeit the detailed activated biochemical pathways have yet to be proven experimentally and remain to be fully understood [26].

With the exception of the cryptochrome photo-induced radical pair as a possible magnetoreceptor in bird sensing, the prevalence of magnetic field effects under physiological conditions *in vivo* has been questioned [27]. Moreover, attempted reproducibility experiments dispute published results for the magnetic field effects in ATP production and B_12_ enzymes [28], [29]. With this caveat in mind, we propose that the widespread presence of free-radical production in cellular metabolism may be influenced to some extent by magnetic fields *via* the SCRPM. In particular, the formation of spin-correlated radical pair states between enzyme FADH^•^ and O_2_^•−^ can be altered by RF magnetic fields [11], [15]. We hypothesize that RF magnetic fields can influence the spin dynamics in free-radical pairs during cellular metabolism, and thereby determine O_2_^•−^ and H_2_O_2_ product yields that are associated with singlet-triplet states in free-radical biochemistries.

To test these ideas, we investigated *in vitro* the effects of magnetic fields on the biological production of intracellular O_2_^•−^ and H_2_O_2_ in rat pulmonary arterial smooth muscle cells (rPASMC). More specifically, the cells were exposed to a controlled 45 μT static magnetic field (SMF) (similar to naturally occurring environmental fields) or a SMF combined with perpendicularly-applied weak RF magnetic fields of 10 μT_RMS_ at 7 MHz. Hereinafter, we will refer to the control group as SMF and the exposed 7 MHz group as RF. To elucidate the link between O_2_^•−^ consumption and H_2_O_2_ production, cellular assays were performed to measure concomitantly O_2_^•−^ and H_2_O_2_ with and without applied RF magnetic fields. Our results suggest that the RF magnetic fields affect not only ROS product distributions but also cellular growth rates. Taken together, this study demonstrates the interplay between O_2_^•−^ and H_2_O_2_ production influenced by RF magnetic fields and underscores the subtle effects of low-frequency magnetic fields on oxidative metabolism, ROS signaling, and cellular growth.

### Spin Biochemistry

There has been considerable theoretical and experimental interest in spin effects that materialize from free radical biological chemical reactions [14], [16], [17], [30]; in particular, the reaction of molecular oxygen activation by reduced flavins, and subsequently, the generation of related active ROS [11], [31]. The Schulten lab have taken the fundamental ideas developed in prior work [16], [17], [23] and applied its significance to an example of biological function *in vivo*, specifically the role of cryptochrome as a magnetoreceptor in the case of avian magnetic sensing [15]. Following the flavin chemistry put forth by Massey [11], Solov'yov postulated a reaction scheme that involves an enzyme-bound neutral flavin FADH^•^ cofactor and O_2_^•−^ as spin-correlated radical pairs in cryptochrome signaling [15]. A similar reaction was also theoretically modeled in the context of glucose oxidase and spin-orbit coupling in FADH^•^/O_2_^•−^ radical pairs [32]–[34]. Hogben *et al.* critically evaluated the feasibility of FADH^•^/O_2_^•−^ spin-correlated radical pair reaction schemes in cryptochrome with emphasis on Zeeman resonances [35]. Based on theoretical grounds, the Oxford Group summarized the various proposed reaction schemes and they expressed doubt about the applicability of different variants of this model in the context of cryptochrome signaling. For the oxidation of FADH_2_ by O_2_ reaction (fig. 1B in Ref. [35]), the authors claim that the reaction is magnetically sensitive in principle, but remain skeptical about the formation of sufficient spin correlation. As a point of departure, we thought that it would be insightful to use this spin biochemistry to develop a better understanding of the general effects of magnetic fields in experimental physical biology. The plan, therefore, was to use cellular assays to demonstrate that metabolic pathways involved in the production of O_2_^•−^ and H_2_O_2_ peroxide are magnetically sensitive reactions, as predicted by spin-correlated radical pair biochemistry, *vide infra*.

**Figure 1:**
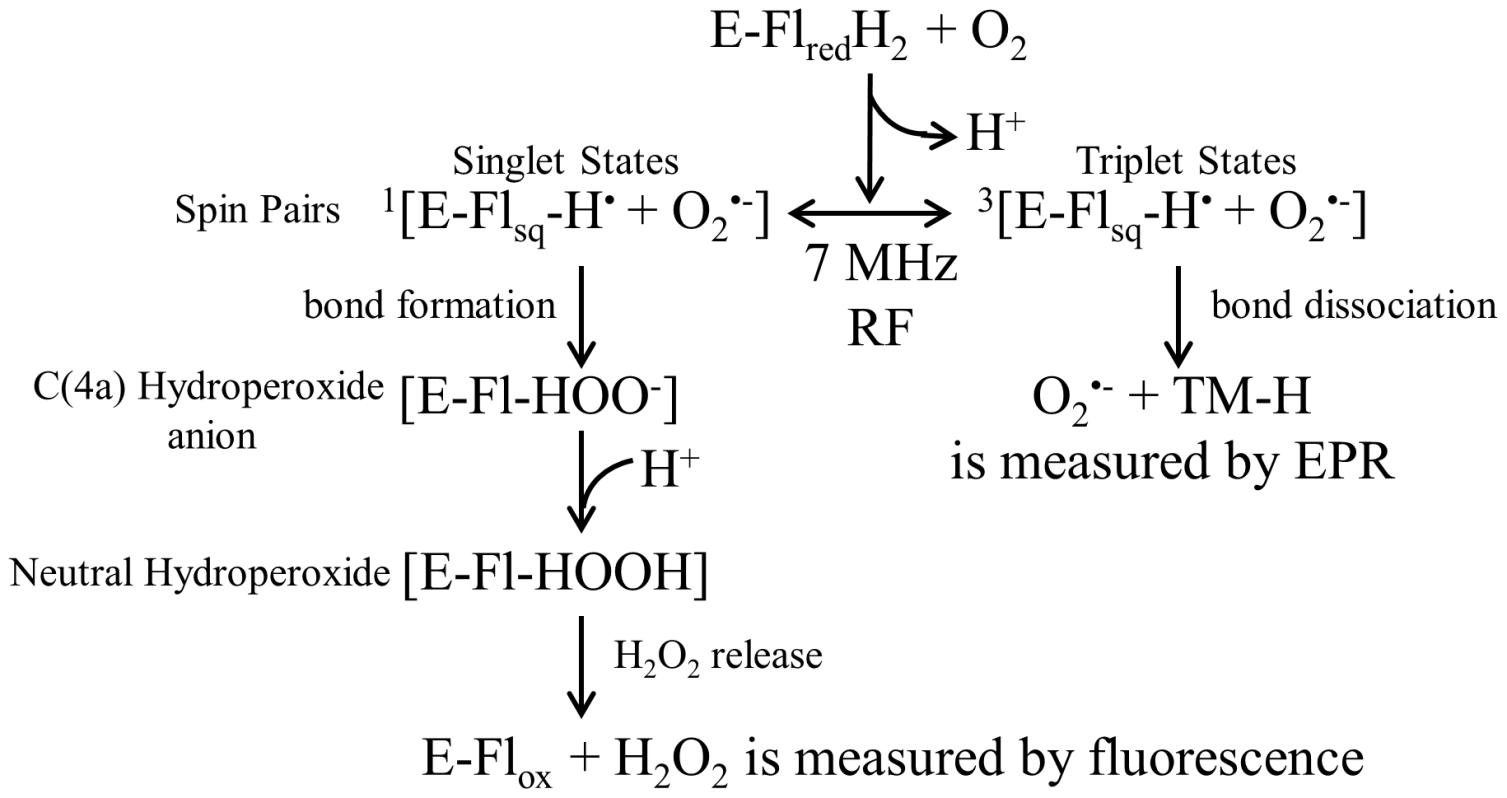
The general reaction scheme involves the spin biochemistry of an enzyme-bound reduced flavin and molecular oxygen. The RF magnetic fields modulate the interconversion rate of singlet-triplet spin correlated radical pairs. This influence disrupts ROS homeostasis, and therefore, the product distributions of H_2_O_2_ and O_2_^•−^, which were measured by separate spectroscopic techniques.

Figure 1 proposes a general reaction scheme that includes the spin biochemistry of an enzyme-bound reduced flavin (FADH_2_) and O_2_. A single electron is transferred from FADH_2_ to O_2_ and produces a FADH^•^ and O_2_^•−^ spin-correlated free radical geminate pair in the triplet state. The 7 MHz (10 μT_rms_) RF magnetic field decouples the flavin hyperfine interactions during spin coherence and thus mediates the interconversion between singlet and triplet states. Electron nuclear double resonance was recently performed on a series of flavin enzymes, and the flavin couplings were found to have anisotropic hyperfine couplings with a bandwidth range of 7–35 MHz [36]. Here, we suggest that spin pairs with the hyperfine resonance frequency of 7 MHz shift the equilibrium of spin pairs into a singlet state, conserving probability and resulting in an increase of singlet products (H_2_O_2_) at the expense of triplet products (O_2_^•−^). In the triplet state, O_2_^•−^ is released from the enzyme “caged” radical pair and can then react with cyclic hydroxylamine spin probes, which is measured by electron paramagnetic resonance (EPR) spectroscopy. The singlet state forms a chemical bond that leads to the formation of flavin C(4a)-hydroperoxide. The addition of a proton forms neutral hydroperoxide and results in the release of hydrogen peroxide, where it is measured by Amplex Red Ultra fluorescence.

The sufficiency for the use of a single oscillating frequency of 7 MHz at the low end of the flavin broadband hyperfine coupling range (7–35 MHz) relies on the premise that one of the spin pairs (O_2_^•−^) is devoid of hyperfine interactions, and thus, the overall hyperfine interaction should be decreased [15], [37]. The putative reduction in hyperfine coupling is greatly dependent on the local environment or range of environments adopted by the spin radical pair, as expected in complex cellular systems. In the context of an applied system, Ritz *et al.* used broadband RF and a single 7 MHz frequency to demonstrate Zeeman (1.3 MHz at 45 μT) and presumably hyperfine (7 MHz) resonance effects, respectively, for an avian magnetic compass [16], [38]. In our work, the goal was to separate Zeeman (1.3 MHz at 45 μT SMF) from hyperfine coupling (7–35 MHz) resonances and attempt to exclusively probe hyperfine coupling energies. Following previously reported literature values for the experimental parameters, the static magnetic field, oscillating frequency, and amplitude were specifically chosen to ensure the most probable success for eliciting a magnetic field response. Many biological molecules exhibit hyperfine splitting constants that range from 0.1–35 MHz [36], [38], and therefore, we propose that 7 MHz frequency most likely influence cellular chemical reactions that involve biomolecules with hyperfine couplings of equal energies [36], [37]. To fully separate the contributions from Zeeman and hyperfine couplings, the magnetic field, frequency, and intensity dependence needs to be evaluated, of which is beyond the scope of this paper.

**Figure 2:**
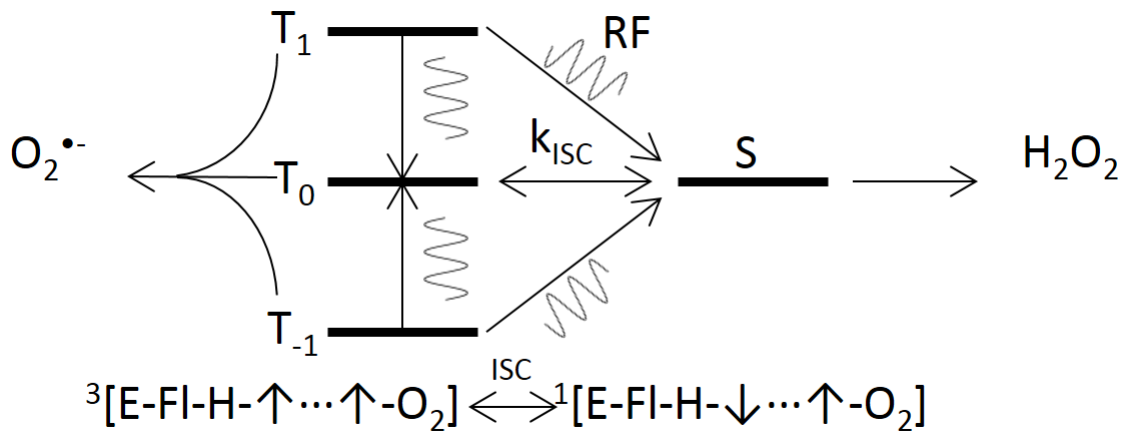
A schematic illustration demonstrates the singlet-triplet transitions that affect the kinetics of the effective intersystem crossing rate (k_ISC_) in the radical pair. The sinusoid lines represent RF-induced transitions, which influence the effective intersystem crossing rate. Radical pairs that commence in the triplet state result in an increase in singlet product yield with concomitant decrease in triplet products, *vice versa*.

To describe the disruption of ROS homeostasis that results in product distribution changes by RF magnetic fields, we employ novel methods that are innovative extensions and applications of product yield-detected magnetic resonance (PYDMR) [39]. A reasonable explanation for the observed results is as follows: (1) the reduced flavin and molecular oxygen form an intermediate spin-correlated free radical pair FADH^•^/O_2_^•−^; (2) the hyperfine transitions of the radical pair modulate spin correlation between them; (3) the intersystem crossing rate between singlet and triplets states of the free radicals is influenced by the magnetic field modulation of the spin correlation; (4) the measured ROS product yields are relatively different because of changes in the singlet and triplet probabilities; hence ROS equilibrium in the cells is fundamentally altered by the experimental oscillating magnetic fields under appropriate conditions. Figure 2 illustrates a simple diagram of RF resonance transitions that can modify product yields for the associated triplet (O_2_^•−^) and singlet (H_2_O_2_) states. It is worth pointing out that the radical pair initial states can be determined by changes in the relative product yield of unperturbed samples (SMF case). If the initial state of the radical pair is in the triplet state, then the triplet products should decrease and the singlet products will increase, *vice versa*.

The generality of the presented reaction and PYDMR schemes are valid for the oxidases but is not restricted to this family of enzymes. Potentially other spin biochemical process might be present in cellular metabolism because the SCRPM does not depend upon any specific chemical identity of the radicals [40]. However, the frequencies, amplitudes, and orientation dependence of the oscillating fields that perturb the radical-pair dynamics depend significantly on the local enzyme (flavin) chemical environments [37], [38], [41]. Therefore, we anticipate that 7 MHz is within the broadband range (0.1–35 MHz) of hyperfine couplings for some flavin enzymes, and thus, the oxidase flavoenzymes should be sensitive to the applied static and oscillating magnetic fields used in this work. For a particular type of enzyme, a full quantum mechanical treatment with the appropriate spin Hamiltonian is needed to calculate, among other theoretical parameters, the Zeeman and hyperfine resonance energies [16], [42]. We would like to stress that our reasoning is a gross simplification of the complex ROS enzymatic processes in redox biochemistry [43] but the simplified general model is sufficient for rationalizing magnetic field effects in oxidative metabolism. One can imagine a branching effect where a few percent change in initial reaction products of an ROS enzymatic process could amplify other biochemical pathways [40].

## Materials and Methods

### Chemical Reagents

Cyclic hydroxylamines 1-hydroxy-4-methoxy-2,2,6,6-tetramethylpiperidine (TM-H), and 5-(diethoxyphosphoryl)-5-methyl-1-pyrroline-N-oxide (DEPMPO) were purchased from Enzo Life Sciences (San Diego, CA, USA). Catalase, polyethylene glycol-conjugated superoxide dismutase (PEG-SOD), diethyldithiocarbamate (DDC), diethylenetriaminepenta-acetic acid (DTPA), diphenyleneiodonium chloride (DPI) and 4-Amino-2,2,6,6-tetramethylpiperidine-1-oxyl (TEMPO) were purchased from Sigma-Aldrich (St. Louis, MO, USA). Paraquat was obtained from Fisher Scientific (Santa Clara, CA, USA). Stock solutions of cyclic hydroxylamine (10 mM) were prepared in argon-purged 0.9% NaCl treated with 0.1 mM DTPA. Stock solutions were prepared daily and kept in an ice bath under argon to avoid autooxidation. Certain commercial equipment, instruments, or materials are identified in this document. Such identification does not imply recommendation or endorsement by the National Institute of Standards and Technology, nor does it imply that the products identified are necessarily the best available for the purpose.

### Cell Culture

Rat pulmonary arterial smooth muscle cells (rPASMC), isolated as previously described [44], [45], were cultured in Dulbecco's Modified Eagle Medium's (DMEM) (Invitrogen, Carlsbad, CA, USA) supplemented with 10% fetal bovine serum (FBS) (ATCC, Manassas, VA, USA) at 37 °C with 5% CO_2_. All animal procedures were approved by the University of Nevada Institutional Animal Care and Use Committee (protocol no. 00365) in accordance with the National Institutes of Health's *Guide for the Care and Use of Laboratory Animals* (1996). The cells were cultured in a 75 cm^3^ flask to expand cell number and to poise the cells until they were confluent. At 2–3 days, the cells reached confluence and the cells were then seeded in 6-well culture plates at a density of 3.5×10^3^ cells/cm^2^.

### Cell-Culture Magnetic Field Exposure System

**Figure 3:**
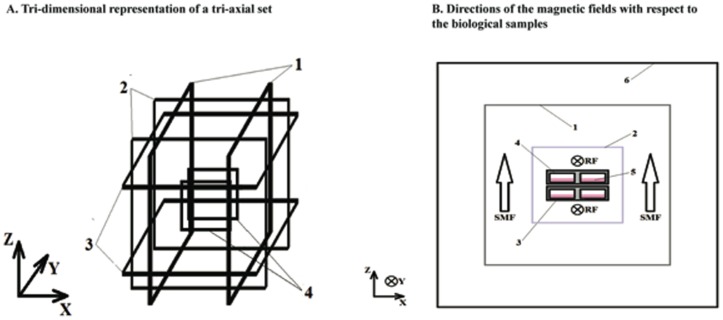
A diagram is shown that represents the experimental apparatus for magnetic field exposure. (**A**) Tri-dimensional representation of the tri-axial set used for controlling static and alternating electromagnetic fields. Square coil pairs in a Helmholtz configuration are geometrically aligned to control the static magnetic field (SMF) and to compensate for fluctuations in the ambient magnetic fields in the (1) horizontal (X) direction, (2) horizontal (Y) direction, and (3) vertical (Z) direction. This diagram also depicts the placement of a square coil in Helmholtz configuration for the generation of RF magnetic fields (4). A Faraday cage was also used in the RF experiments to surround the setup to minimize RF reflections, but it is not shown in this diagram for clarity. (**B**) This figure depicts the directions of the magnetic fields with respect to the biological samples. (1) A tri-axial set of square coils in Helmholtz configuration for SMF generation in all 3 dimensions; (2) square coils in Helmholtz configuration for RF generation in the horizontal (Y) direction; (3) an individual 6-well plate; (4) individual wells; (5) culture medium; and (6) a Faraday cage.

The initial background SMF inside the incubators, due largely to the earth's magnetic field, varied from 25 to 60 μT as measured with a gauss meter (IDR-321, Integrity Design, VT, USA) in all 3 axes, and therefore required tri-axial compensation to establish a uniform pre-set SMF in the volumes designated for culture plates within the incubator. For these experiments, two tri-axial sets of square coils were constructed in a Helmholtz configuration (Fig. 3). The first set allowed for the simultaneous exposure of three 6-well cell culture plates as a control to a SMF of 45 μT. The second set served to expose cells to a SMF of 45 μT and to perpendicularly-applied 7 MHz magnetic fields. In both cases, the 45 μT SMF was oriented perpendicular to the plane of growth of the cells. Sham exposures were conducted only for DC magnetic fields because an equivalent sham exposure for the RF fields, an energized RF coil in the control incubator with a shielded RF magnetic field, is a significant technical challenge. The experimental exposure included both groups placed within separate tri-axial coils containing a single Helmholtz loop RF loop in separate incubators. The RF coil was not energized for the control SMF and was energized for the RF group.

Each square coil (25 cm each side) consisted of 20 turns of 22 AWG enamel-coated copper wire. Each pair of square coils was axially aligned and separated by 12 cm in order to achieve the Helmholtz configuration [46]. Each pair of coils in the Helmholtz configuration was individually driven by a power supply (HP 6205C Dual, Hewlett-Packard, Palo Alto, CA). Resistive circuitry was fed in a twisted pair in order to achieve the necessary compensatory SMF in the desired direction. The SMFs were adjusted accordingly at the isocenter of each tri-axial set as measured by a gauss meter for each axis. A 1-turn square coil (12.5 cm side) in Helmholtz configuration was built inside one of the tri-axial sets in order to superimpose magnetic fields in the RF band also with 22 AWG enamel-coated copper wires (Fig. 3). The geometric center of this RF coil was aligned with that of the tri-axial set used for SMF compensation. A function generator (HP33120A, Hewlett-Packard, Palo Alto, CA) established the 7 MHz magnetic signal, and the magnitude recorded in the culture-designated volume was 10±3 μT (RMS) after power amplification. A grounded Faraday cage (40 cm side) surrounded the RF tri-axial set in order to contain the signal within the volume of the SMF-RF exposure and to exclude deleterious background signals. The RF signal was measured with a circular sensor composed of 2 turns of 22 AWG enamel-coated copper wires, 1.5 cm in radius, which were connected directly to an oscilloscope *via* a twisted pair feeding a coaxial cable. The applied electric current was measured by the voltage drop across a 2 Ω resistor in series with the RF coil.

The background time-varying magnetic field was measured at the center of the tri-axial sets while inside the incubator in the location where the experiment was to be performed with a gauss meter (IDR-210, Integrity Design, VT) in all 3 axes. The measurements performed resembled previous observations [47], where the dominant spectral magnitude was recorded at 60 Hz and was below 2 μT for all cases. The temperature and CO_2_ concentration were maintained at 37 °C and 5% respectively with Binder CB-150 incubators. The environmental parameter variance was minimal during the experiments. The incubators were utilized exclusively for these experiments and were not opened for the duration of the exposures.

### Cell Proliferation and Viability Experiments

The effects of the magnetic field exposure on cellular proliferation were determined directly by counting the number of cells after each termination point. For the cell counting assay, 6-well plates were seeded at a concentration of 3.5×10^3^ cells/cm^2^ for rPASMC. The initial time (*t_0_*), when magnetic exposure began, was defined as 24 h after initial cell seeding. At each termination point (*t_1_* =  day 2, *t_2_* =  day 3), cells from 3 wells were counted twice with a hemocytometer (VWR, San Francisco, CA, USA) with concomitant cell viability assessed by the trypan blue exclusion method. A typical cell-viability experiment involved cells that were gently harvested and mixed with 0.4% trypan blue solution (Invitrogen). The resulting cell suspension was counted under a phase-contrast inverted microscope. Viable cells with intact cell membranes excluded the dye and were counted with the hemocytometer.

### Fluorometric Detection of H_2_O_2_ Production

Cellular H_2_O_2_ production was measured with the horseradish peroxidase-linked Amplex Ultra Red (HRP-AUR; Invitrogen) fluorometric assay. Cells were seeded as described in the cell-proliferation experiments (see above) and then were exposed to SMF and RF magnetic fields for the duration of the experiment. At *t_1_* and *t_2_*_,_ medium was aspirated and cells were washed with PBS plus 100 μM DTPA and incubated for 2 h with DMEM containing 2% FBS, 10 μM AUR and 0.2 units/ml HRP. Resorufin fluorescence was collected on a Gemini fluorescence microplate reader (Molecular Devices, Sunnyvale, CA). Cellular number, protein content, and resorufin fluorescence were measured at the same termination points. H_2_O_2_ production was normalized to total protein content and cell count. H_2_O_2_ calibration curves with HRP-AUR under the same RF magnetic field strengths showed no differences compared to SMF controls, thus demonstrating that RF fields do not interact with the H_2_O_2_ detection assay.

### Superoxide Measurements

All rPASMC intracellular superoxide measurements were performed in DMEM media containing 2% FBS and 0.1 mM DTPA. For the superoxide assay, cells were seeded at a concentration of 3.5×10^3^ cells/cm^2^ and kept under SMF and RF for 3 days (*t_2_*) without disturbance. On day 3, cells (∼90% confluent) were washed with phosphate buffered saline (PBS) that included 0.1 mM DTPA, and then the cells were incubated for 60 minutes at 37 °C in 220 μL of DMEM, 2% FBS, 0.1 mM DTPA and 0.5 mM TM-H. The experiments were conducted in the presence and absence of Paraquat (200 μM), PEG-SOD (50 units/ml), DDC (10 μM), DPI (10 μM), as indicated below. Thereafter, the plates were briefly kept on ice until the buffered cells were harvested with a scraper and were snap-frozen in liquid nitrogen (100 μL each). At this point, the cellular samples were ready for low-temperature EPR spectroscopy and were scanned for the TM-H paramagnetic signal.

### Electron Paramagnetic Resonance (EPR) Spectroscopy

**Figure 4:**
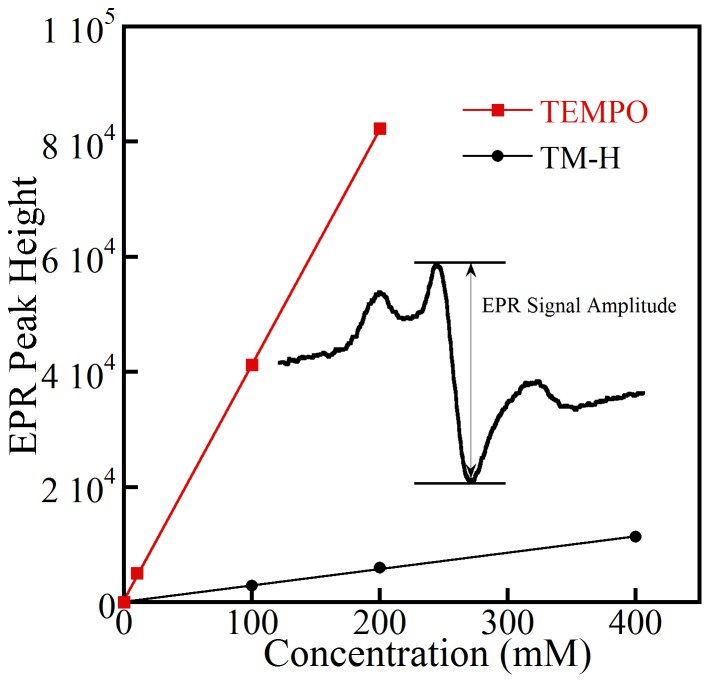
EPR signal of nitroxide free radicals that were formed by reacting cyclic hydroxylamines spin probe T-MH in the xanthine oxidase-superoxide-generating system containing xanthine oxidase 10 mU/ml, xanthine (100–400 μM), and DTPA (0.1 mM). The efficiency of TM-H spin probe was compared to peak heights of standard TEMPO nitroxide concentrations. For the same concentrations of reacted xanthine and TEMPO, TM-H peak height slope was 1/14 as large.

Continuous-wave (CW) EPR spectra of the cyclic hydroxylamine TM-H with and without rPASMC were recorded at ∼9.2–9.3 GHz on a Bruker X-band spectrometer with a Bruker ER4102ST TE_102_ cavity and a liquid nitrogen-cooled gas-flow system. The spectra were collected under the following operating conditions: 108 K, 0.5 mT modulation amplitude at 100 kHz modulation, a microwave power of 2 mW, 128 ms time constant, and averaging five 15.0 mT wide scans. A standard curve of 4-hydroxy-TEMPO (Sigma Aldrich), with concentrations ranging from 10–200 μM, was produced to determine quantitatively the relative concentration of the TM-H nitroxide free-radical from the signal amplitude [13], [48], Figure 4. Superoxide radical was initially measured by EPR in a cell-free system of cyclic hydroxylamine TM-H (0.5 mM) and the xanthine oxidase-superoxide-generating system that contained xanthine oxidase (10 mU/ml), xanthine (100–400 μM), and DTPA (0.1 mM). The cyclic hydroxylamine free radical concentration was calculated by comparing center peak height (Fig. 4 inset) ratios of the EPR signal to the center peak-height ratios for the signal from a TEMPO standard (10–200 μM) recorded under the same conditions [13]. Because the cellular environments contain multiple paramagnetic metal centers that can influence the relaxation rates of the spin probes, comparison of TEMPO and T-HM is only qualitative. The background control signal of extracellular media, taken at the same time points as for the RF cell systems, was subtracted from all TM-H cyclic hydroxylamine samples.

### Protein Determination

To calculate the protein concentration of the cell population, cells from the 6-well plate O_2_^•−^ and H_2_O_2_ assays were washed twice with 1 ml of PBS buffer. Then 250 μl of Radio-Immunoprecipitation Assay (RIPA) buffer was added to the dish and incubated on ice for 5 min. The lysate was kept frozen until used further. The lysate was thawed on ice and centrifuged at 8,000×g for 10 min. 20 μl of the supernatant was added to 200 μl of bicinchoninic acid (BCA) working reagent (Pierce BCA Protein Assay Kit) in a 96-well plate. The plate was incubated at 37°C for 30 min and the absorbance was measured at 562 nm. The protein concentration was calculated from a standard curve.

### Statistical Analysis

Statistical analysis was performed by use of 1-way analysis of variance (ANOVA) with a minimal confidence level of 0.05 for statistical significance. Each experiment was performed at least 3 times with a minimum of three samples per termination point (day 2 or day 3) per experiment. The data shown constitute representative samples of the experiments performed. All the experiments were performed semi-double blind. For both EPR and optical spectroscopy, the samples were prepared and coded by one researcher, the data were collected by another researcher (blind) and then the final results were normalized by protein concentration and cell count by the original preparer.

## Results

### Weak 7 MHz Magnetic Fields Enhance Cell Proliferation of rPASMC

**Figure 5:**
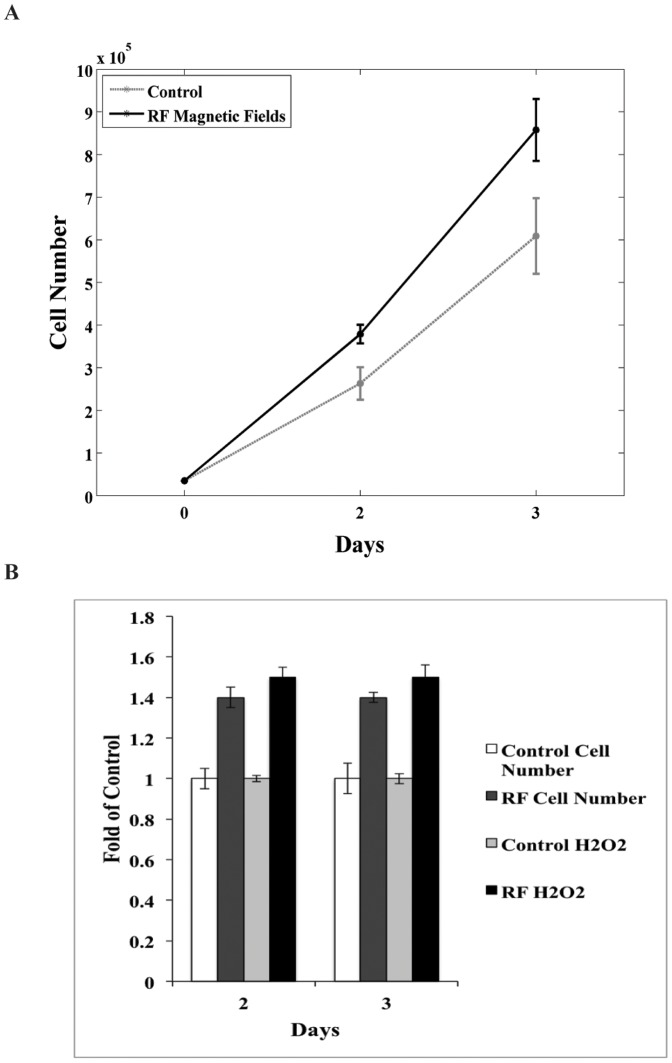
The RF-induced cellular proliferation and hydrogen peroxide production is shown for rPASMC, and is greater compared to control samples. After the cells reached >90% confluence, the RF electromagnetic field was turned on, Day 0. (A) RF 7 MHz magnetic fields enhanced cell growth by ∼40% on day 2 and ∼45% on day 3 as determined by direct count and trypan blue exclusion method. Data are a representative sample of 3 independent experiments. (B) H_2_O_2_ production was also measured to compare to cell proliferation. RF electromagnetic fields increased production of H_2_O_2_ by ∼50% on days 2 and 3 of exposure as determined by AUR assay. Data are normalized to SMF control.

rPASMC cell numbers progressively increased during the course of the experimental culture period (1–3 days). Enhanced cell proliferation was observed with continuous applied 45 μT SMF and 7 MHz 10 μT_RMS_ magnetic fields compared to the control group with only 45 μT SMF. The RF magnetic fields enhanced cellular proliferation by up to 40% on day 2 and 45% on day 3 in proportion to the SMF control group (Fig. 5a). Cell viability, assessed by the trypan blue exclusion method, showed no significance in staining between SMF and RF groups. Overall, the cellular-growth curves indicate that the applied RF magnetic fields had no effect on cell viability compared to the control but indeed had a statistically significant effect on cellular growth profiles.

### Combination of Magnetic Fields Enhances H_2_O_2_ Production in rPASMC

In an attempt to link cell proliferation to H_2_O_2_ production for SMF and RF exposure, cell number and H_2_O_2_ were measured simultaneously at the same termination points for days 2 and 3. Figure 5b compares SMF control cell numbers to the applied RF cell numbers and the SMF control H_2_O_2_ to the RF H_2_O_2_. rPASMC exposed to RF magnetic fields produced ∼50% more H_2_O_2_ compared to the SMF control for both termination points. The results here demonstrate an increased amount of H_2_O_2_ for both termination days for RF samples with a similar amount of H_2_O_2_ produced for each day in proportion to the increased cell count.

Catalase was added as a negative control at a concentration of 40 units/ml. Addition of external catalase suppressed the RF magnetic field effects on H_2_O_2_ production, where catalase brought RF magnetic fields levels of H_2_O_2_ production similar to control levels (data not shown). Diethyldithiocarbamate (DDC 10 μM) and polyethylene glycol-superoxide dismutase (PEG-SOD 50 U/ml) were used as positive controls and showed a decrease in H_2_O_2_ and an increase in H_2_O_2_ production, respectively (data not shown). The use of PEG-SOD demonstrates that the origin of H_2_O_2_ production is likely located at an intracellular source that is leached out of the cell and detected in the extracellular media.

**Figure 6:**
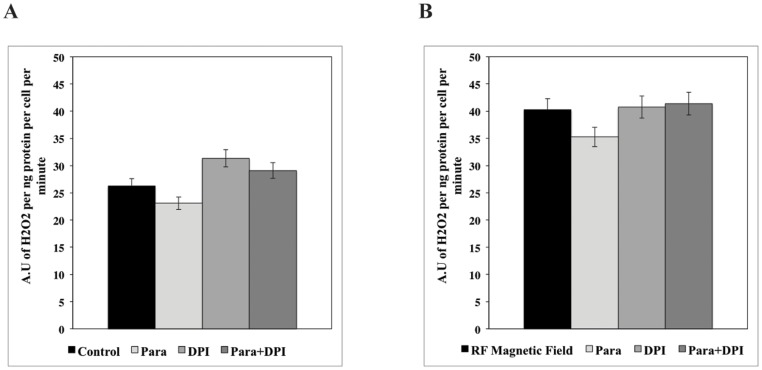
RF magnetic fields increase H_2_O_2_ production in rPASMC independently of Paraquat and DPI. (**A**) The Paraquat control (200 μM) slightly decreases H_2_O_2_ production, while DPI (10 μM) slightly increases H_2_O_2_ compared to the SMF control cells (**B**) RF magnetic fields show no preferential effects on Paraquat/DPI-induced H_2_O_2_ production. RF magnetic field overall enhances H_2_O_2_ production in rPASMC by 30 to 40% as seen by (B) RF vs. (A) SMF control. Data are a representation of three independent experiments.

Paraquat and DPI were used to induce oxidative stress in rPASMC *in vitro*. Photomicroscopy showed that exposure of cells to paraquat (200 μM) and DPI (10 μM) did not change cellular morphology or viability during two hours of xenobiotic treatment. Paraquat slightly decreases H_2_O_2_ production, whereas DPI had a small increase in H_2_O_2_ production as compared to the SMF control samples on rPASMC *in vitro* (Fig. 6a). However, the collective dataset (Fig. 6a,b) demonstrates that the RF magnetic fields modulate the general H_2_O_2_ production trend independent of Paraquat and DPI. The baseline for the RF magnetic fields and xenobiotics are roughly 30 to 40% larger than the SMF control groups. Overall, these results suggest that the RF magnetic fields induce an increase of H_2_O_2_ production but show no preferential effects on Paraquat/DPI-induced H_2_O_2_ production.

### Detection of O_2_^•−^ by EPR and Cyclic Hydroxylamine Spin Probes

**Figure 7:**
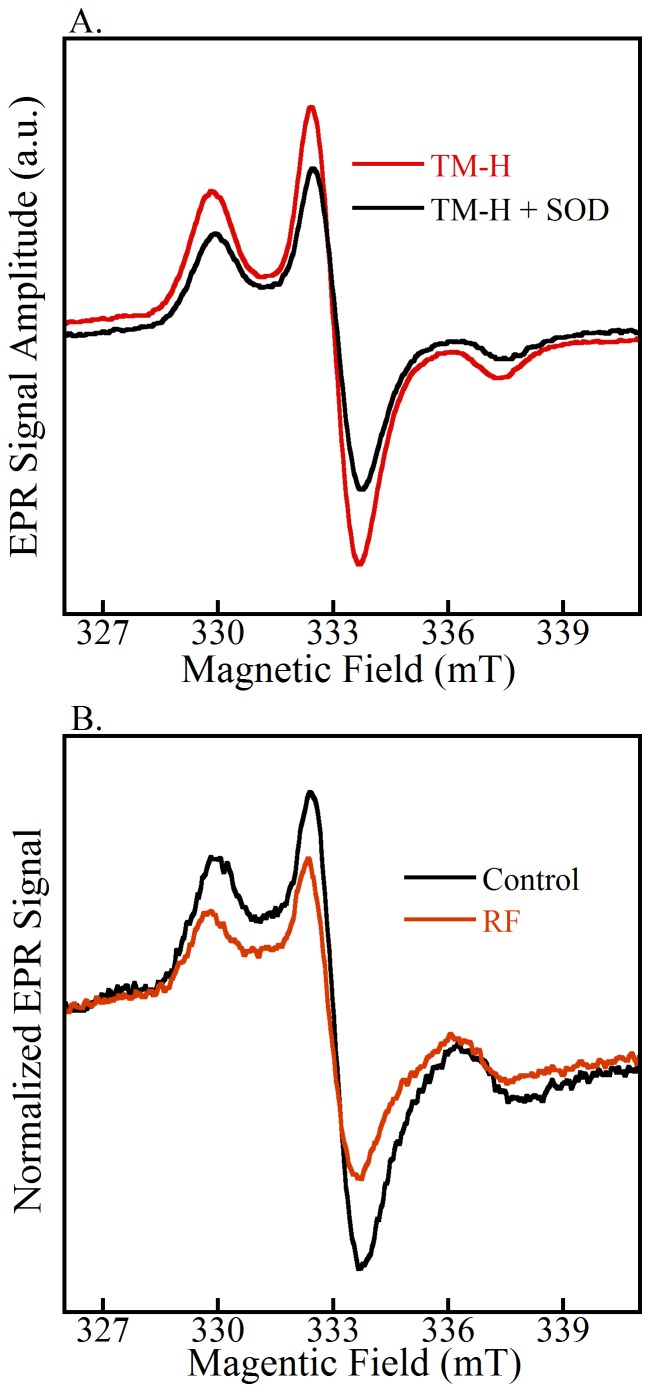
EPR spectra of cyclic hydroxylamines in cell free controls and an example of RF experiments for the detection of superoxide. (A) PEG-SOD (50 U/ml) inhibits the EPR signal by up to 40% in a cell-free xanthine/xanthine oxidase system. (B) Control and RF normalized EPR spectra. TM-H spin probe reacts with intracellular superoxide to give a nitroxide free-radical that is detectable by EPR. The RF samples have a lower EPR signal intensity compared to control, indicative of a lower intercellular superoxide concentration.

To determine the qualitative efficacy of the hydroxylamine spin probe, TM-H was reacted with O_2_^•−^ produced in a cell-free system by xanthine oxidase [48]. Figure 4 shows the linear relationship in TM-H middle-peak EPR signal amplitude as a function of xanthine concentration (100–400 μM) and demonstrates the fidelity of cyclic hydroxylamine as a spin probe for superoxide detection. A similar experiment was carried out by use of spin-trap DEPMPO (10 mM), and the result produced no visible EPR signal above noise in the experimental time frame. The redox chemistry of cyclic hydroxylamines proved to be more effective for reacting with O_2_^•−^, and the radical signal intensity was larger and lasted substantially longer than traditional spin traps (data not shown) [48]. To demonstrate that O_2_^•−^ flux can be controlled and subsequently detected by TM-H, SOD (50 U/ml) was added to the cell-free xanthine/xanthine oxidase system. The accumulation of O_2_^•−^ was blocked by SOD, and the corresponding EPR signal amplitude of TM-H was diminished by 40% (Fig. 7a).

In the rPASMC systems, Figure 7b demonstrates comparison of O_2_^•−^ detection with the spin-probe TM-H for SMF and RF magnetic fields. The data processing includes the background subtraction of the TM-H of the media, and the signal amplitude was normalized to cell count and protein concentrations. The EPR spectra are shown for typical data of normalized cyclic hydroxylamine-nitroxide free radicals measured in cell samples for at least three independent experiments.

### Superoxide Consumption Is Enhanced by External RF Magnetic Fields

**Figure 8:**
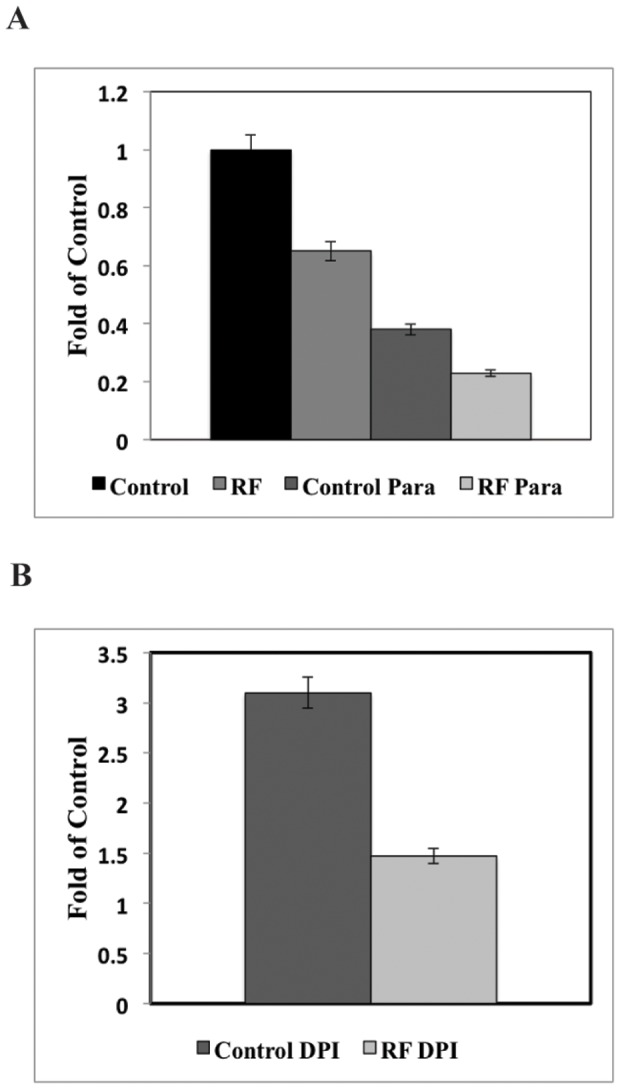
RF electromagnetic fields and xenobiotics had significant effects on cellular detected superoxide. (**A**) RF initially decreased the amount of detected superoxide compared with SMF control by 40%. In the Paraquat (200 μM) samples, O_2_^•−^ was initially suppressed by 60% compared with SMF control and the effects were enhanced by RF magnetic fields by 50% compared with the Paraquat control. (**B**) In the DPI (20 μM) increased O_2_^•−^ production by 200% compared with SMF control, whereas RF DPI superoxide was decreased by 50% compared with DPI control. The data shows typical results observed in at least three independent experiments.

In order to better understand how RF magnetic fields mediate the production of O_2_^•−^, EPR of cyclic hydroxylamines were compared to the control SMF and RF exposed cells. Figure 8 shows that RF magnetic fields significantly reduce the amount of O_2_^•−^ concentration by 40% in rPASMC, compared to SMF. The reduction in detected basal O_2_^•−^ concentration implies either an increase in consumption or a decrease in production of O_2_^•−^. The corresponding decrease in O_2_^•−^ species with the accompanying increase in H_2_O_2_ implies a RF-induced modulation in the distribution of ROS products.

Chemical species that control the production of superoxide were used to explore a possible mechanism of action of the RF magnetic field. Paraquat, which normally induces O_2_^•−^ production, was observed to inhibit O_2_^•−^ concentrations by 60% in the SMF control compared to basal cells, and RF magnetic fields further enhanced this suppression by 50% compared to Paraquat control. DPI (20 μM) increased O_2_^•−^ production by 200% compared to SMF control, whereas RF DPI superoxide was decreased by 50% compared to DPI control. PEG-SOD 50 units/ml decreased intracellular O_2_^•−^ concentrations whereas the SOD inhibitor DDC (10 μM) increased O_2_^•−^ concentrations for SMF, as expected (data not shown). RF exposure further enhanced O_2_^•−^ suppression by PEG-SOD and further increased proliferation of O_2_^•−^ by DDC. We interpret the inhibition of the TM-H EPR signal by PEG-SOD as demonstrating that the spin-probe targets intracellular superoxides, in agreement with previous observations [48]. These results suggest rPASMC O_2_^•−^ production was diminished by Paraquat and PEG-SOD, and was enhanced by DPI and DDC under SMF. Despite increase or decrease of O_2_^•−^ by the xenobiotic samples, all samples showed an enhanced decrease in measured O_2_^•−^ by RF magnetic fields.

## Discussion

The goal of this work was to determine RF magnetic field effects on ROS production in oxidative metabolic processes that potentially involve spin-correlated radical pairs. The strategy was to assay O_2_^•−^ and H_2_O_2_ concentrations with and without applied RF magnetic fields and to correlate the product distributions with cell proliferation rates. The SCRPM was used to rationalize the changes in ROS product distribution by PYDMR with the application of RF magnetic fields. We hypothesized that the ROS product distributions would be influenced by RF-mediated singlet-triplet interconversion. The spin-pair model was postulated to involve an enzyme-bound semiquinone flavin and superoxide free-radicals with singlet products forming H_2_O_2_ and triplet products forming O_2_^•−^. Our novel methodology and experimental results indirectly support the mechanism of spin biochemistry between flavoenzymes with oxygen and can begin to address some of the outstanding questions of flavin and oxygen reactions; e.g., release of O_2_^•−^ by some flavoenzymes [14].

Solv'yov *et al.* and others recently proposed a link between O_2_^•−^ and H_2_O_2_ production for cryptochrome magnetoreception that involves spin-correlated radical pairs between O_2_^•−^ and FADH^•^ [15]–[17], [49]. For ROS production in normal cellular metabolism, we suggest a different reaction scheme originally proposed by Massey that is based on the activation of molecular oxygen (O_2_) by (E - Fl_red_H_2_) reduced flavoenzymes [11], [34]:
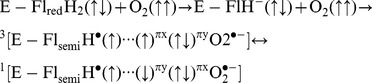



This reaction mechanism is initialized with a proton charge-transfer process that poises anionic FADH^−^ for an electron transfer to triplet molecular oxygen O_2_ to form a spin-correlated radical pair; i.e., FADH^•^ and O_2_^•−^ (Fig. 1). The spin-correlated radical pair is initially in the triplet state because there is no change of spin state during the electron-transfer process [34], [35]. In a weak SMF, the hyperfine mechanism populates all four spin states in a quasi-steady state population having the ratios S_0_:T_+_:T_0_:T_-_ = 2/9∶2/9∶3/9∶2/9 [50], [51]. Therefore, the equilibrium occupation probability that the radical pair will remain in one of the three triplet states is 7/9 (2/9 for the singlet state) because the SMF is below the hyperfine energies and all four states remain coupled. RF magnetic fields influence the recombination rate of singlet-triplet interconversion that determines reaction product yields, a process analogous to the quantum and chemical Zeno effects [52], [53]. During spin coherence, RF magnetic fields decouple the flavin-hyperfine interactions and thus modulates the interconversion rate, which changes the equilibrium value between singlet- and triplet-state probabilities, Fig. 2 [34]–[36], [39]. The spin-correlation duration must persist long enough (>1 μs) to allow the RF magnetic fields to have significant effects on reaction productions yields [35], a situation that has been questioned for radical pairs free in solution but is plausible within an enzyme pocket [35], [54].

We hypothesized that 7 MHz (10 μT_rms_) applied RF magnetic fields decouple hyperfine interactions, rather than Zeeman resonances (1.3 MHz at SMF 45 μT), between O_2_^•−^ and FADH^•^ free-radical pairs [16], [35], [38]. With an applied 7 MHz RF magnetic field, the probability of the spin-pair being that of the singlet state increased at the expense of probability occupying triplet-state configurations, Figs. 1 and 2. Therefore, the amount of triplet state O_2_^•−^ released from the enzyme was expected to *decrease*, a result that was measured by the spin-probe TM-H and EPR spectroscopy, Fig. 7b. Spin-pairs in the singlet state form a chemical bond that results in the flavin-C(4a) hydroperoxide anion species, a quasi-stable product that is considered an activated form of oxygen and can be converted, among other reaction pathways [11], [14], into neutral hydroperoxide with an addition of a proton [14]. The neutral hydroperoxide dissociates to form oxidized FAD and H_2_O_2_, where released H_2_O_2_ was measured by the fluorescence assay. The singlet products (H_2_O_2_) from the spin-pair reaction are diamagnetic, which is normally spin-forbidden for the triplet state products due to spin conservation during the electron transfer [15], [34], [35]. The increase in the population of singlet states from the spin-radical pair resulted in the observed increase in H_2_O_2_ production and an accompanying increase in O_2_^•−^ consumption; i.e., a decrease in detected O_2_^•−^.

Although our work does not directly probe the specific enzymes involved in the spin-radical pair mechanism *per se*, we attempted to measure a connection between RF magnetic field effects on O_2_^•−^ consumption and H_2_O_2_ production by rationalizing our results through the postulated reaction mechanism, Fig. 1. The plan was to develop a broader understanding of the spin biochemistry associated with RF magnetic fields and the general effects of RF magnetic fields in biology. In biological systems, the controlled production of ROS is considered to have important mediating effects in physiological and pathological processes [55]–[57]. The interplay between ROS generation and feedback systems are thought to regulate many biological processes with ROS products as signaling agents. In particular, O_2_^•−^ and H_2_O_2_ redox regulation in the Nox pathways have been linked to many cellular processes, including cell differentiation and proliferation [58].

A RF magnetic field-induced increase in cellular proliferation with accompanying increase in H_2_O_2_ production for the rPASMC is shown in Figure 5. The RF magnetic fields increased cell growth by ∼40% and increased H_2_O_2_ production by ∼50%, as compared to controls. This result provides evidence that the increase in H_2_O_2_ production by RF magnetic fields affect one or more underlying ROS-producing enzymatic pathways that regulate cellular growth. We suggest that the RF effects of H_2_O_2_-increased production create an oxidative stress environment, and thus trigger a growth mechanism in rPASMC cells similar to increased Nox4 expression [59]. H_2_O_2_ has been recognized as a secondary messenger in redox thiol-based redox switches [60], [61] and sensitive thiol transduction [62], where an upregulation of Nox4 leads to an increase in H_2_O_2_ signaling that can lead to increased cell proliferation in smooth muscle cells [63], [64].

To explore a more direct role of RF magnetic field-effects on NADPH oxidase activity, we introduced Paraquat and DPI in an attempt to isolate NADPH oxidase ROS reaction pathways. In the H_2_O_2_ assays, the salient feature in these results is that the collective baseline for all the measured samples was substantially larger (30–40%) with applied RF magnetic fields (Fig. 5) when compared to SMF groups. H_2_O_2_ measurements showed minimal effects of the xenobiotics in SMF and exhibited elevated-H_2_O_2_ production similar to the control-applied RF sample. For the O_2_^•−^ assays, Paraquat showed a decrease in O_2_^•−^ production of 40% compared to that of basal cells, which is in contrast to normal induced-ROS production for this chemical, Figure 8 [65]. DPI exhibited an increase in O_2_^•−^ production compared to that of basal cells. RF showed further enhanced Paraquat and DPI suppression of O_2_^•−^ concentration by 40% and 58%, respectively, compared to SMF baselines. In the absence of xenobiotics, a reasonable interpretation is that O_2_^•−^ acts as a precursor for H_2_O_2_ production, and the RF magnetic fields enhance superoxide consumption, which leads to the observed increase in H_2_O_2_ production. However, difficulty arises in the interpretation of the effects of Paraquat and DPI on the NADPH oxidase ROS pathway in conjunction with applied RF magnetic fields. We observed a slight correlation with Paraquat reduction in O_2_^•−^ and H_2_O_2_ trends; however, we expect a significant increase in H_2_O_2_ with RF and DPI. In other words, there are no statistically-significant changes in H_2_O_2_ production with the addition of Paraquat, DPI, and the applied RF magnetic fields that can be related to a spin-pair mechanism. In light of these observations, we cannot rule out RF effects on NADPH oxidase activity, but the results indicate that no spin-pair biochemistry effects in NADPH oxidases can be attributed to the inhibitors.

We speculate that the flavoenzymes, including oxidases and monooxidases, are the oxidative metabolic enzymes that are most likely influenced by spin biochemistry and RF exposure at the experimental static magnetic field, amplitude, and frequency. In particular, glucose oxidase, as an alternative to the Nox enzyme systems, is flavoenzyme that could be probed for spin biochemistry by measuring ROS product distributions as a function of media glucose concentrations rather than the use of inhibitors [34]. Another interpretation of the overall results can be explained by RF effects from another ROS source, such as mitochondria, that serves as magnetoreceptors to produce ROS *via* SCPRM. We are tempted to suggest that there is an influence in the cross-talk from mitochondria to NADPH oxidases by RF-induced changes in ROS production within mitochondria [56], but direct experimental evidence is needed to validate this assertion. Experiments with other chemical inhibitors are in progress to isolate the specific metabolic pathway that is associated with RF magnetic effects in metabolism.

We hypothesized that O_2_^•−^ and FADH^•^ spin-correlated radical pairs are the source of the ROS production mediated by RF magnetic fields during oxidative metabolism. Our experimental ROS reaction product distributions can be understood by the SCPRM in the hyperfine coupling energy regime with these static and oscillating magnetic fields [38]. The influence on ROS production that leads to changes in cell proliferation seems to be a result of elevated H_2_O_2_ levels that act as secondary messaging molecules. RF orientation, magnetic field amplitude, and broadband frequency experiments are needed to further validate the spin radical pair mechanism in biochemical processes and to probe other spin-correlated radical pair mechanisms and molecules that might be present in oxidative metabolism and ROS generation. The current experiments did not include a true “off-resonance” experiment where the application of the RF frequency is not on-resonance with the hyperfine couplings. Our experiments describe the case where there is an “on” or “off” applied 7 MHz RF magnetic field effect where we hypothesize hyperfine coupling resonance. Investigations into the various magnetic field parameters and their dependences will provide a more complete description of the magnetic field effect profile in biochemical and cellular processes, and further biochemical and cellular engineering protocols can be developed in this emerging area of research.

## Conclusion

The central goal of this work was to measure RF magnetic effects on oxidative metabolism that produces ROS and to confirm the changes in reaction products yields within the context of a rational spin-biochemistry model. The results presented here demonstrate a link between superoxide consumption and hydrogen peroxide production that was mediated by RF magnetic fields, which was delineated in the context of the SCRPM. The ROS product distribution is thought to occur by decoupling hyperfine energies that modulate singlet-triplet states and thus determine reaction-product yields. A secondary effect of increased hydrogen peroxide production is believed to create oxidative stress on the cells that resulted in an increase in cell proliferation. Because of the indirect measurement of spin effects in metabolism, we cannot rule out other possible mechanisms for magnetic field effects in ROS production and cell proliferation.

With the near ubiquity of radiated non-ionizing magnetic energy present in the environment, understanding the non-thermal effects from broadband RF exposure on oxidative metabolism would be clearly beneficial and is important from a public-safety standpoint [66]–[68]. In contrast to the spin-pair mechanism, specific absorbed radiation (SAR) measurements of macroscopic tissue heating represent a naive approach to bio-magnetic RF-interactions because it ignores nanoscale physics and spin chemistry, which can potentially have profound biological effects. In addition to safety concerns or potential benefits, new areas of technological development in medical and electronic interfacing with biological systems could be pursued by identifying, understanding, and monitoring metabolic processes that are influenced by RF magnetic fields. More generally, a deeper understanding of spin biochemical processes is possible by elucidating magnetic fields effects in biology and by implementing spin radical pair mechanism principles in broader areas of experimental design. The methodology presented here formulates one route that can address the connection between the atomic level and cellular level, where cellular magnetic field responses transcend the quantum/classical interface.
