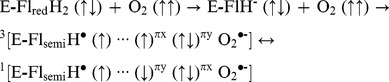# Correction: Spin Biochemistry Modulates Reactive Oxygen Species (ROS) Production by Radio Frequency Magnetic Fields

**DOI:** 10.1371/journal.pone.0101328

**Published:** 2014-06-20

**Authors:** The PLOS ONE Staff

There is an error in the scheme shown in the “Discussion” section. Please refer to the correct scheme below: